# Continuous Monitoring with Implantable Loop Recorders After Cryoballoon Ablation: Impact on Atrial Fibrillation Recurrence and Therapeutic Management in Era of Artificial Intelligence

**DOI:** 10.3390/jcm14092932

**Published:** 2025-04-24

**Authors:** Rosario Foti, Michele Di Silvestro, Giuseppe Campisi, Sergio Conti, Giuseppe Picciolo, Carlo Cardì, Marco Barbanti, Giulia Rapisarda, Antonio Parlavecchio, Giuseppe Sgarito

**Affiliations:** 1 PO San Vincenzo, Azienda Sanitaria Provinciale Messina, 98123 Messina, Italy; 2 PO Umberto I, Azienda Sanitaria Provinciale Enna, 94100 Enna, Italy; micheledisilvestro@live.it (M.D.S.);; 3 PO Giovanni Paolo II, Azienda Sanitaria Provinciale Ragusa, 97100 Ragusa, Italy; 4 Azienda di Rilievo Nazionale ad Alta Specializzazione Ospedale Civico-Di Cristina-Benfratelli, 90127 Palermo, Italy; 5 University of Iowa Health Care, Department of Internal Medicine, Division of Cardiology, Section of Clinical Cardiac Electrophysiology, The Carver College of Medicine, University of Iowa, Iowa, IA 52242, USA; 6 Istituto di Ricovero e Cura a Carattere Scientifico Bonino Pulejo, 98124 Messina, Italy; 7 Department of Medicine and Surgery, Università degli Studi di Enna “Kore”, 94100 Enna, Italy; 8 Istituto di Ricovero e Cura a Carattere Scientifico Istituto Mediterraneo Trapianto e Terapia ad alta Specializzazione, UPMC Palermo, 90133 Palermo, Italy; giuseppesgarito@gmail.com

**Keywords:** atrial fibrillation ablation, cryoballoon ablation, implantable loop recorder, artificial intelligence, AF management

## Abstract

**Objectives**: Atrial fibrillation (AF) is the most common sustained arrhythmia associated with stroke, heart failure, and increased mortality. Due to its efficacy and safety, cryoballoon ablation (CBA) is widely accepted for rhythm control; however, long-term AF recurrence remains a challenge. Continuous monitoring with implantable loop recorders (ILRs) enhanced by artificial intelligence (AI) can detect both symptomatic and asymptomatic episodes, potentially optimizing patient management. This analysis assessed the long-term effectiveness of CBA in maintaining sinus rhythm and investigated the role of ILR-guided monitoring in enhancing therapeutic decisions. **Methods**: Data from 91 patients with paroxysmal or persistent atrial fibrillation (AF) who underwent pulmonary vein isolation using cryoballoon ablation at four Italian centers between April 2022 and April 2024 were analyzed. All patients received an insertable loop recorder (ILR) before or during hospitalization for ablation, allowing for the continuous remote monitoring of arrhythmias. Baseline demographics, procedural details, AF occurrence, AF burden (calculated as the total duration of all AF episodes occurring within a day and categorized by episode duration), therapeutic adjustments, and the effect of artificial intelligence (AI) on data processing were evaluated. **Results**: The cohort’s average age was 62.4 years, with 24.2% of participants being female. Physician-confirmed AF recurrence was noted in 26.7% of patients at 12 months and 49.5% at 24 months. The device data indicated a daily AF burden of ≥6 min in 47.2% at 12 months, with 25.9% surpassing 1 h. AI algorithms decreased false-positive alerts by 21%, resulting in an estimated saving of 19 clinician hours. In patients with pre-ablation ILR data, the median AF burden significantly decreased from 7% to 0.2% (*p* = 0.017). ILR-guided monitoring affected treatment adjustments, leading to the discontinuation of antiarrhythmic therapy in 36 patients and redo ablations in 8. **Conclusions**: Continuous ILR monitoring, combined with AI-driven analysis, enables the detection of AF recurrences and burden, thereby facilitating timely therapeutic adjustments.

## 1. Introduction

Atrial fibrillation (AF) is the most prevalent sustained cardiac arrhythmia, associated with a higher risk of stroke, heart failure, and overall mortality [1,2,3]. Catheter ablation remains one of the most commonly used treatments for rhythm control, with cryoballoon ablation (CBA) and other techniques widely applied due to their effectiveness and favorable safety profile [1,4,5,6,7]. However, achieving long-term success presents a challenge, as AF recurrence can occur despite initially successful ablation. Continuous monitoring with implantable loop recorders (ILRs) has transformed post-ablation follow-up by providing comprehensive, long-term arrhythmia detection beyond symptomatic episodes [8,9,10]. Unlike conventional intermittent monitoring, ILRs enable a more accurate assessment of AF recurrence, including asymptomatic episodes and AF burden, which may have significant clinical implications [11]. The recent integration of artificial intelligence (AI) into arrhythmia detection algorithms has further improved the utility of continuous monitoring. AI-driven systems can effectively distinguish true AF episodes from false positives caused by noise, oversensing, or other non-arrhythmic events. This technological advancement maximizes the potential of ILRs by significantly reducing clinicians’ time reviewing data, allowing them to focus on clinically relevant episodes. Moreover, AI enhances the accuracy of AF burden assessment, ensuring that therapeutic decisions rely on precise and reliable information, which is vital for optimizing patient outcomes.

In this analysis, we aimed to assess both the long-term efficacy of CBA in maintaining sinus rhythm and the role of continuous monitoring in optimizing patient management. By leveraging ILR data, we evaluated AF recurrence in terms of daily AF burden. We investigated the impact of continuous monitoring on clinical decision making, specifically how ILR-guided data influenced therapeutic adjustments made by physicians, including modifications to anticoagulation and antiarrhythmic drug therapy. Furthermore, we estimated the effects of these new AI algorithms on clinical outcomes and workflow efficiency, providing a comprehensive evaluation of their role in enhancing patient care through personalized and timely interventions.

## 2. Methods

### 2.1. Patient Cohort and Data Collection

This analysis included all consecutive patients diagnosed with paroxysmal or persistent atrial fibrillation (AF) who underwent pulmonary vein isolation (PVI) via cryoballoon ablation (CBA) at four Italian cardiological centers between April 2022 and April 2024. All patients who agreed to be monitored with an ILR, including remote monitoring, were included. Patients were excluded based on the following criteria: (1) permanent AF; (2) unstable angina or acute myocardial infarction within three months; (3) the need for or prior cardiac surgery within six months; (4) contraindication to treatment with oral anticoagulants; and (5) severe chronic renal or hepatic impairment. All patients received an implantable cardiac monitoring device (Reveal LINQ I and II, Medtronic Inc., Minneapolis, MN, USA) before or during hospitalization for the ablation procedure (on the same day or the day after the procedure). This device was placed subcutaneously near the left parasternal area. Patients were introduced to a home monitoring system that automatically transmits nightly data from the device to a telemedicine platform. The monitoring system (Carelink, Medtronic Inc.) powered by the AccuRhythm™ AI platform (Medtronic, Inc.) can identify the daily AF burden lasting at least six minutes, along with other arrhythmic events. Follow-up was conducted through the remote monitoring system and routine hospital visits, adhering to each institution’s standard clinical protocols under the One Hospital ClinicalService umbrella. The initiative represents a forward-looking evaluation of CBA procedures performed within Italian cardiology departments. The One Hospital ClinicalService program is a comprehensive clinical data repository formed by a network of cardiac facilities. This consortium aims to enhance the implementation of therapeutic interventions in real-world clinical settings through the collaborative aggregation and analysis of patient data. All research activities adhered to the ethical guidelines outlined in the Declaration of Helsinki and received approval from the local institutional review board with all participants providing informed written consent [12,13].

### 2.2. Data Collection

Comprehensive baseline assessments were conducted, encompassing demographic data, arrhythmia classification and history, cardiac comorbidities documentation, and cardiac function evaluations. Procedural metrics were recorded, including Total Procedure Time (measured from the initial groin puncture to the final withdrawal of the catheter), Fluoroscopy Time (the total duration of fluoroscopic imaging during the procedure), and Left Atrial Dwell Time (the time from transseptal puncture to the removal of the catheter from the left atrium). Additionally, any complications that arose during or after the procedures were systematically captured and recorded for all participants.

During the follow-up period, personnel dedicated to telemedicine carefully reviewed all episodes transmitted automatically by the monitoring devices. Patients were contacted directly when necessary to ensure data completeness, and manual transmissions were added as needed. Each AF event was manually classified based on electrogram (EGM) data. After the index AF ablation, a 90-day blanking period was observed during which any atrial arrhythmia detections were not considered recurrences; thereafter, the first AF episode, confirmed by a physician regardless of duration, was defined as the primary efficacy endpoint of the procedure. To characterize AF recurrences, AF daily burden was documented. AF daily burden was automatically calculated by the device as the sum of all AF episodes occurring within a day and was classified into five groups based on duration: ≥6 min, ≥1 h, ≥6 h, ≥24 h, and ≥7 days. Percent AF burden was calculated as the percentage of total time spent in atrial fibrillation over the entire observation period (after a 3-month blanking period). Physicians received AI-generated reports through the AccuRhythm™ AI platform, which provided an assessment of potential false positives. These reports helped streamline the review process by highlighting the number of true arrhythmic events, filtering out noise or artifacts, and optimizing clinical decision making. Moreover, all modifications to pharmacological therapy during the follow-up period were systematically recorded, particularly changes in antiarrhythmic and anticoagulant medications. These adjustments were evaluated in relation to arrhythmic events to assess their impact on patient management.

### 2.3. Statistical Analysis

Continuous variables are presented as mean ± standard deviation when normally distributed or otherwise as median and interquartile range (IQR) values. Categorical variables are reported as counts and percentages. Comparisons between groups were undertaken using Wilcoxon’s rank sum test, while categorical variables were compared with the Chi-square or Fisher’s exact test, as indicated. Variables were also graphically represented, using box plots for continuous variables and bar charts for categorical variables, whenever applicable. All statistical tests were conducted at a two-sided significance level of 0.05. The pre-post comparison of AF daily burden utilized the Wilcoxon’s signed rank test. Event-free survival probability was estimated using the Kaplan–Meier method. A Cox proportional hazards regression analysis was performed to identify clinical and demographic predictors of atrial fibrillation (AF) recurrence. Both univariate and multivariate models were constructed, and variables with clinical relevance or a *p*-value < 0.1 in the univariate analysis were included in the multivariate model. Statistical analysis was performed using the SAS software, version 9.4 (SAS Institute Inc., Cary, NC, USA).

## 3. Results

### 3.1. Patient Population

Demographic and baseline clinical characteristics are summarized in Table 1. A total of 91 patients underwent their first cryoballoon ablation (CBA) and received an implantable loop recorder (ILR) before or within three months of the index procedure. The mean age was 62.4 ± 9.7 years, and 24.2% (22/91) of the cohort were female. Most patients presented with paroxysmal AF (82.4%, 75), while 15.4% (14) had persistent AF and 2.2% (2) had long-standing persistent AF. A history of atrial flutter was reported in 7.6% (6) of cases. The mean time from the first atrial arrhythmia episode to ablation was 49.4 ± 50.3 months. Notably, 47 patients had previously failed two or more antiarrhythmic drugs (AADs). Regarding symptom burden, most patients were classified as EHRA class 2 or 3, indicating moderate to severe symptomatology. Heart failure was present in 17.8% of patients who were in NYHA class 2 or 3 at the time of the procedure. In terms of comorbidities, 59.3% (54/91) had hypertension, and 54.9% (50/91) had valvular heart disease, predominantly mitral valve disease (52.7%, 48). Diabetes was observed in 10.9% (10) of patients and chronic kidney disease in 5.4% (5). The mean CHA_2_DS_2_-VASc score was 1.9 ± 1.4, with 26.6% (21/79) of patients scoring 2 and 6.3% (5/79) scoring ≥ 5.

### 3.2. Procedural Data

The CBA procedure had an average total duration of 87.1 ± 22 min, with a fluoroscopy time of 19.7 ± 8 min. The key procedural data are summarized in Table 2. In total, 77.3% of patients began the procedure in sinus rhythm, and 98% completed the CBA while remaining in sinus rhythm. Among the 91 patients treated, only 1 experienced a periprocedural complication, specifically phrenic nerve palsy, which resolved by the 7-month follow-up. Table 2 presents procedural data and acute complications. In 18 (19.7%) patients, the ILR was inserted on average 159.8 ± 315.9 days before the CBA procedure. In the remaining 73 patients, the device was implanted before hospital discharge after the CBA procedure. No complications were reported. All patients were provided with the CareLink system and received instructions to enable daily remote monitoring of arrhythmias.

### 3.3. AF Recurrence

**Figure 1:**
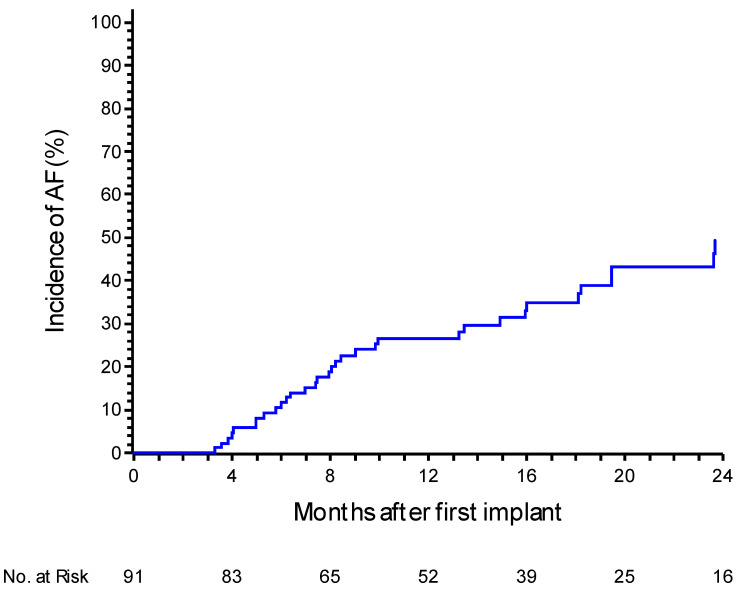
A Kaplan–Meier curve for atrial fibrillation recurrence events after the 3-month blanking period, with a medical review. The curve depicts the probability of recurrence over time based on clinical evaluations by the physician.

**Figure 2:**
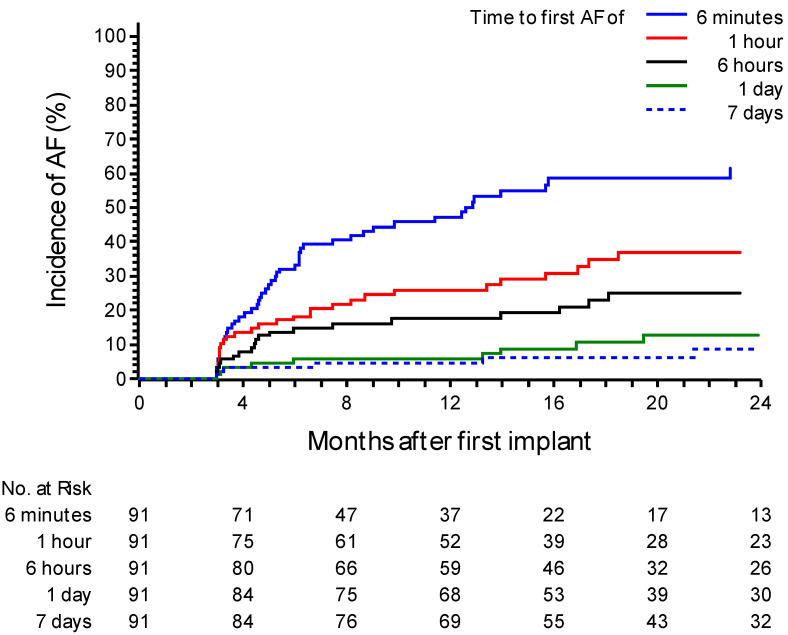
A Kaplan–Meier curve for atrial fibrillation (AF) recurrence events post ablation after the blanking period based on device data. The curve distinguishes patients based on different daily durations of AF, including groups with a daily AF duration of ≥6 min (“≥6 min”), ≥1 h, ≥6 h, ≥24 h, or 7 consecutive days. The graph shows the probability of recurrence over time based on the AF burden recorded by the device.

During a mean follow-up of 21.7 ± 11 months, 33 patients (36.2%) experienced an episode of AF confirmed by medical staff. The incidence of confirmed AF at one year was 26.7% (95% CI: 18.4–37.7%). Notably, only 45% (15 out of 33) of patients were symptomatic. At 24 months, the incidence increased to 49.5% (95% CI: 36.9–63.7%) (Figure 1). In 25 patients (75%), the episodes of AF confirmed by the medical staff had a daily burden of at least 6 min but less than 1 h. In the remaining eight patients (25%), the episodes lasted at least 1 h. Analyzing the daily AF burden recorded solely by the device without AI filtering, the incidence of AF lasting at least 6 min was 47.2% (95% CI 37.1–58.4%) at 12 months. The incidence was 25.9% (95% CI 17.8–36.7%) for a daily AF burden of at least 1 h, 17.5% (95% CI 10.9–27.4%) for a burden of at least 6 h, and 5.7% (95% CI 2.4–13.2%) when a daily AF burden of at least 24 h was observed. In instances where the AF burden exceeded 7 days, the incidence reached 4.6% (95% CI 1.7–11.7%) (Figure 2).

Considering only the subgroup of patients with paroxysmal AF, at 12 months, the incidence of a daily AF burden lasting 6 min is 46.5% (95% CI 35.7–58.9%). Figure S1 shows the incidences in the population of patients with paroxysmal AF only.

In patients with an AF daily burden of at least 6 min (*n* = 49), the median percentage AF burden was 1.1% (IQR: 0.7–7.6), which included a median percentage AF daily burden of 1.0% (0.7–7.1) in patients with paroxysmal AF at baseline and 6.2% (IQR: 0.5–12.7) in those with persistent or long-standing persistent AF. Interestingly, the number of days with an AF daily burden greater than 6 min was 5.5 (2.0–47.0) across the entire population. Specifically, this was 5.0 (2.0–46.0) days in patients with paroxysmal AF and 8.0 (1.0–93.0) days in those with persistent AF. Table 3 shows the percentages of patients who experienced AF episodes of varying durations throughout the follow-up period and the median time to the event.

Considering the blanking period, 23 patients had an AF daily burden of over 6 min. Among these, 21 patients experienced a second episode during the follow-up. In a subgroup of 18 patients with ILRs implanted prior to ablation, the AF burden significantly decreased from 7% to 0.2% post ablation (*p* = 0.017). To explore factors associated with AF recurrence, univariate and multivariate Cox regression analyses were performed. In the univariate analysis, diabetes (HR 5.41, 95% CI: 1.21–24.13; *p* = 0.027) and a history of stroke/TIA (HR 18.74, 95% CI: 5.19–67.64; *p* < 0.001) were significantly associated with recurrence. No significant associations were found for age, sex, AF type (paroxysmal vs. persistent), hypertension, heart failure, or cardiovascular disease (all *p* > 0.05) (Table 4).

### 3.4. Artificial Intelligence Impact on AF Detection

In our analysis, devices recorded 384 AF episodes. AI algorithms eliminated 102 false episodes, reducing the number of AF episodes that required manual verification by 21%. Of these, 11 (10.7%) were false positives due to noise, while the remaining episodes (91, 89.3%) were characterized by a fast atrial rhythm, chaotic rhythm, or bigeminy. According to the literature data [14], the median time for manually reviewing each alert is approximately 11.3 min. Consequently, implementing AI algorithms in the analyzed population resulted in estimated time savings of 19 h for clinicians, calculated as (102 × 11.3)/60 min.

### 3.5. Patient Management

At baseline, 88.6% (81) of patients were on anticoagulant therapy, while 87.9% (80) were receiving class I or III AADs. At the 3-month follow-up, these percentages decreased to 70.9% (64) for antiarrhythmic therapy and 78.2% (71) for anticoagulation. During a median follow-up of 21 months, 60.0% (55) of patients remained on antiarrhythmic therapy, and 56% (51) continued anticoagulant treatment. Following the transmission report of AF events in the 49 patients, a telephone contact was initially made with 22 patients to assess their general health status and symptoms related to AF. For the majority, an outpatient visit was requested (43 patients). Five patients independently requested a visit to the AF clinic. Anticoagulant therapy was discontinued in 30 patients. In 36 patients, antiarrhythmic medication was stopped after the first 6 months due to the absence of AF episodes in the post-blanking period. However, in 11 patients, a new antiarrhythmic medication was reintroduced following AF recurrence. Of these, 6 patients (55%) were symptomatic and reported episodes of AF with a median overall burden of 2.0%. The remaining five patients (45%) were asymptomatic, with a median burden of 0.3%. In two patients with a daily burden >24 h, cardioversion was performed, and eight patients with a recurrence of symptomatic AF underwent a redo ablation. Of these, two had a history of persistent or long-standing persistent AF and six of paroxysmal AF. Notably, no patient sought emergency care or was hospitalized for decompensated heart failure.

## 4. Discussion

As emphasized in current guidelines, AF ablation is primarily guided by patient symptoms [1,2]. The decision to pursue repeat ablations depends mainly on the severity, frequency, and impact of these symptoms on quality of life [1]. However, continuous monitoring devices have enhanced our understanding of AF by detecting episodes—particularly brief or asymptomatic—that may not correspond with patient-reported symptoms [8,9]. For instance, in a study of patients with paroxysmal or persistent AF who had implanted pacemakers for unrelated indications, AF was detected in 88% of patients over 19 ± 11 months of follow-up, with more than one-third experiencing asymptomatic episodes lasting over 48 h [15]. Similarly, Tondo et al. reported that up to 45% of patients with AF recurrences detected by ILRs were asymptomatic [16]. Post-ablation, symptom perception may also evolve due to changes in atrial tissue or neural pathways or to placebo effects.

Importantly, a recent meta-analysis [17] examined the association between symptom status and clinical outcomes in 217,850 patients with AF, demonstrating no significant differences in the risk of all-cause mortality, cardiovascular mortality, thromboembolism, stroke, hospitalization, or myocardial infarction between symptomatic and asymptomatic individuals. The recent literature emphasizes the necessity of continuous monitoring for detecting asymptomatic or subclinical AF, which can significantly influence treatment decisions and patient outcomes [18,19]. Device-based monitoring, particularly with ILRs, provides a comprehensive view of AF burden, enabling earlier interventions and potentially lowering the risks associated with untreated AF. The LINQ AF study [20] demonstrated how success rates can vary based on the method of rhythm assessment, with single-procedure success rates ranging from 46% to 79% according to the evaluation criteria. Similar findings were previously published in a sub-study of the STAR-AF 2 Trial [21]. While scientifically rigorous, continuous recurrence analysis may overestimate failure rates by including all post-blanking AF episodes, even those that are infrequent or clinically insignificant. This limitation is especially relevant in persistent AF cases, where prolonged healing phases can distort interpretations of treatment success.

In our analysis, focusing solely on device-detected data, 47.2% of patients experienced AF recurrence within one year, which is defined as a daily AF burden exceeding 6 min. However, when medical professionals reviewed these episodes—considering ECG data, single episode duration, and patient-specific factors—only 26.7% of patients were classified as having physician-confirmed AF recurrences. This rate closely mirrors the incidence of an AF daily burden exceeding 1 h, observed in 25.9% of patients. This alignment suggests that shorter, device-detected episodes may not always signify clinically meaningful recurrences. By integrating the device data with expert clinical evaluation, our approach offers a more accurate assessment of true AF recurrence, reducing the overestimation often associated with device-only monitoring.

Our findings align with previous studies that utilized continuous monitoring. In a cohort of 102 patients with paroxysmal AF treated with CBA, 65.7% remained free from any atrial tachyarrhythmia (AT)/AF episodes at 12 months post ablation, accounting for a 3-month blanking period. At two years, 59.3% maintained freedom from recurrence [22]. Similarly, the EARLY-AF trial by Andrade et al. compared CBA with AADs in patients with paroxysmal AF, reporting a 57.1% freedom from AT/AF at one year post CBA—aligning closely with our results [23]. The CIRCA-DOSE trial, a multicenter, randomized study, evaluated 346 paroxysmal AF patients undergoing conventional radiofrequency (CFRF) ablation or two different CBA protocols. The primary outcome was the time to the first recurrence of symptomatic or asymptomatic AT/AF episodes lasting more than 30 s, detected via various monitoring methods, including ILRs. While recurrence rates after a single procedure were similar (~53%) across groups, the study highlighted a nearly 99% reduction in AF burden from baseline as a key indicator of ablation success. Notably, patients with ILRs implanted before ablation experienced a reduction in AF burden from 7% to 0.2% post procedure, reinforcing the importance of continuous monitoring in optimizing outcomes [24].

Our analysis estimated the significant role of AI in enhancing the efficiency of AF detection and management. The AI algorithms were trained using a supervised learning process that involved ECG recordings from a diverse population collected using multiple device types. This diverse dataset, including over 1.4 million adjudicated episodes, allowed the AI to generalize effectively and avoid overfitting to any specific biases. The algorithms were validated using real-world data, with high sensitivity (99.2%) and specificity (82%) for AF, and similarly high performance for Pause episodes. This robust training and validation process ensures that the AI can reliably detect AF and Pause episodes, even in the context of varied signal characteristics and patient demographics [25,26].

The AI algorithms integrated into the monitoring system eliminated 102 false AF episodes from a total of 384 indeterminate episodes sent for clinician review. This corresponds to a 21% reduction in transmissions requiring manual verification, substantially decreasing the workload for healthcare providers. Based on the literature data [14], implementing AI resulted in estimated time savings of 19 h for clinicians. Reducing false positives alleviates the burden on clinical staff and minimizes patient anxiety associated with unnecessary clinical follow-ups. Integrating AI into continuous monitoring systems demonstrates a promising approach to improving the accuracy and efficiency of post-ablation patient management, ultimately enhancing patient outcomes and optimizing healthcare resources.

In our cohort, ILR data significantly influenced clinical management. The detection of asymptomatic AF in almost half of the cases enabled timely interventions, including adjustments to antiarrhythmic or anticoagulant therapy. Real-time data supported personalized treatment strategies, potentially reducing recurrence risk. For example, two patients with an AF daily burden exceeding 24 h underwent cardioversion, while eight required repeat ablation procedures, highlighting how continuous monitoring can guide clinical decisions. In addition, asymptomatic AF seems to be predominantly detected in elderly males, with relevant comorbidities and with a more circular transverse thoracic shape section. Conversely, individuals with symptomatic AF are commonly younger, with reduced comorbidity burden, and with a concave-shaped chest wall conformation. Accordingly, considering the impact of comorbidities and the influence of anthropometrics, continuous monitoring with ILR seems to be more appropriate in this subset of patients [27,28].

Our findings add to the growing evidence supporting the integration of continuous monitoring in AF management, particularly after ablation. ILRs provide a more accurate measure of AF recurrence and enhance clinical decision making, ultimately improving patient outcomes. However, challenges persist in interpreting device data and integrating these insights into routine practice. Future research should aim to refine AF burden thresholds and assess the long-term impact of personalized management strategies informed by continuous monitoring.

### Limitations

This observational, multicenter analysis involves a relatively small sample size (*n* = 91), which may limit the generalizability of the findings. The modest cohort size may not fully capture the clinical variability observed in larger or more diverse populations. Additionally, the mean follow-up duration of 21.7 months, while sufficient to provide mid-term insights, may not adequately assess the long-term efficacy of CBA, especially considering the progressive nature of AF. Late recurrences might have gone undetected. Furthermore, evaluating AF episodes was somewhat subjective, as treating physicians determined whether specific episodes were clinically relevant. This could introduce inter-operator variability in data interpretation and therapeutic management as there were no shared management protocols among the centers. Clinical characteristics, demographics, symptomatology, and burden are all factors that influenced patient care. A much larger study could better guide patient management.

## 5. Conclusions

In conclusion, combining CBA with continuous monitoring via implantable devices enhanced by AI represents a promising strategy for managing AF. This approach provides critical insights into arrhythmia recurrence and its impact on patients, fostering a more personalized, effective, and timely treatment paradigm. Integrating these advanced technologies into routine clinical practice could significantly improve AF treatment outcomes.

## Figures and Tables

**Table 1:** Baseline characteristics.

Baseline Characteristics	Total (*N* = 91)
**Demographic Data**	
Age at First Ablation (yrs)	62.4 ± 9.7
Gender (Female)	24.2% (22)
**Cardiovascular History**	
BMI (Kg/m^2^)	27.4 ± 3.4
Symptoms—Palpitations	82.4% (77)
Symptoms—Other than palpitations	95.6% (87/91)
**EHRA Score (Continuous)**	2.4 ± 0.7
** Type of Atrial Fibrillation**	
Paroxysmal	82.4% (75)
Persistent	15.4% (14)
Long-Standing Persistent	2.2% (2)
**History of Flutter**	7.5% (6)
**Months from first Atrial Arrh. Episode (Continued)**	49.4 ± 50.3
**First-Line Patients**	2.4% (2)
**Number of Tested/Failed AAD—1**	45.8% (41)
**Number of Tested/Failed AAD—2+**	51.8% (48)
**NYHA**	
No HF	82.8% (75)
1	3.4% (3)
2	10.9% (10)
3	3.4% (3)
4	0.0% (0)
**History of Stroke/TIA**	7.7% (7)
**Hypertension**	59.3% (54)
**Diabetes**	10.9% (10)
**IRC**	6.5% (5)
**Any Valve Disease**	54.9% (50)
**Mitral Valve Disease**	52.7% (48)
**Aortic Valve Disease**	3.3% (3)
**Tricuspid Valve Disease**	2.2% (2)
**Pulmonary Valve Disease**	0.0% (0)
**CHA_2_DS_2_-VASc**	
0	18.7% (17)
1	25.3% (23)
2	25.3% (23)
3	17.6% (16)
4	7.7% (7)
≥5	5.5% (5)
**Echo Parameters**	
Left Ventricle Ejection Fraction (%)	55.4 ± 6.7
Left Atrium volume	47.0 ± 15.4
**Drug Therapy**	
Beta-Blockers	62.6% (57)
Diuretics	25.2% (23)
Ace Inhibitors	48.3% (44)
Class I or Class III AAD	87.9% (80)
NAO or TAO	88.6% (81)

**Table 2:** Procedural data.

Procedural Characteristics	Total (*N* = 91)
**Procedure Duration (min)**	87.1 ± 22.3
**Fluoroscopy Duration (min)**	19.7 ± 8.7
**Acute Success Rate**	100.0%
**Pre-Ablation Rhythm**	
Synus	77.3% (71)
AF	21.6% (19)
Flutter	1.1% (1)
**Cardioversion**	25.3% (23)
**Post-Ablation Rhythm**	
Synus	97.7% (89)
AF	2.3% (2)
**Left Atrium Dwell Time (min)**	48.0 ± 27.2
**ILR Implant**	
ILR Implant Before CBA procedure	19.7% (18)
ILR Implant During CBA Hospitalization	80.3% (73)
**Acute Complications**	
**Transitory Diaphragmatic Paralysis**	1.1% (1)

Legend: AF: atrial fibrillation; CBA: cryoballoon ablation; ILR: implantable loop recorder.

**Table 3:** The percentage of patients with atrial fibrillation (AF) events recorded by implanted loop recorders, stratified by different durations of AF daily burden. The median time to event for each group is also shown.

Daily AF Duration Time to Event (Months)	Total (*N* = 91)
6 min AF Time to event	49 (53.8%)
	7.3 ± 6.3
1 h AF Time to event	29 (31.9%)
	8.3 ± 7.7
6 h AF Time to event	19 (20.9%)
	7.1 ± 5.3
1 day AF Time to event	9 (9.9%)
	9.2 ± 6.6
7 days AF Time to event	6 (6.6%)
	8.5 ± 7.5

**Table 4:** Univariate and multivariate Cox regression analyses for predictors of atrial fibrillation recurrence. Hazard ratios (HRs) with 95% confidence intervals (CIs) and *p*-values are reported. Variables included in the multivariate model were selected based on their clinical relevance and statistical significance in the univariate analysis. Diabetes and a prior history of stroke or transient ischemic attack (TIA) emerged as independent predictors of AF recurrence.

	Univariate		Multivariate	
Variable	HR (95% CI)	*p*-Value	HR (95% CI)	*p*-Value
Age (Continuous)	0.98 (0.94–1.01)	0.156		
Gender (Male)	0.77 (0.34–1.77)	0.541		
Paroxysmal AF	0.72 (0.28–1.87)	0.498	0.76 (0.29–2.05)	0.593
History of Heart Failure	1.50 (0.46–4.90)	0.505		
Diabetes	5.41 (1.21–24.13)	0.027	8.23 (1.76–38.57)	0.007
History of Stroke/TIA	18.74 (5.19–67.64)	<0.001	22.43 (5.94–84.70)	<0.001
Hypertension	0.67 (0.35–1.31)	0.244		

## Data Availability

The data are available upon reasonable request.

## Supplementary Materials

The following supporting information can be downloaded at: https://www.mdpi.com/article/10.3390/jcm14092932/s1, Figure S1: Kaplan-Meier curve for atrial fibrillation (AF) recurrence events post-ablation after the blanking period, based on device data only in the subgroup of patients with paroxysmal AF.
