# Supplementary material for: Continuous Monitoring with Implantable Loop Recorders After Cryoballoon Ablation: Impact on Atrial Fibrillation Recurrence and Therapeutic Management in Era of Artificial Intelligence
Source: J Clin Med. 2025 Apr 24;14(9):2932. doi: 10.3390/jcm14092932 (PMC12072361; doi:10.3390/jcm14092932)
Supplement: Supplementary file 1 [file jcm-14-02932-s001.zip › jcm-3567212-supplementary.pdf]

Figure S1: Kaplan-Meier curve for atrial fibrillation (AF) recurrence events post-ablation after the blanking period, based on device data only in the subgroup of patients with paroxysmal AF. The curve distinguishes patients based on different daily durations of AF, including groups with daily AF duration of ≥ 6 minutes ("≥ 6 min"), ≥ 1 hour, ≥ 6 hours, ≥ 24 hours, or 7 consecutive days. The graph shows the probability of recurrence over time based on the AF burden recorded by the device

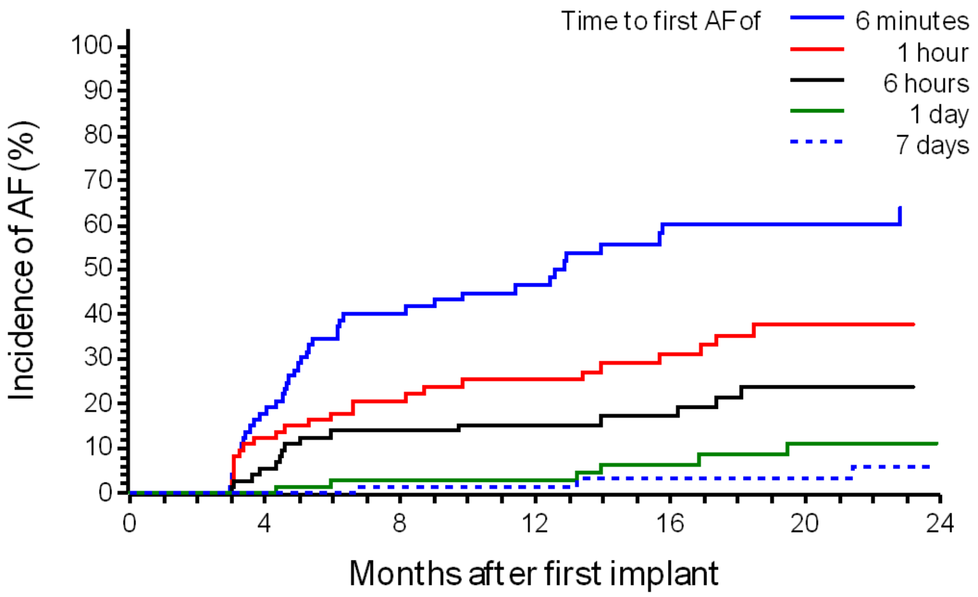

No. at Risk							
6 minutes	75	59	39	31	18	14	10
1 hour	75	63	52	45	35	25	20
6 hours	75	68	57	52	42	29	23
1 day	75	72	65	60	48	35	26
7 days	75	72	66	61	50	39	28
